# Molecular Epidemiology of O139 *Vibrio cholerae*: Mutation, Lateral Gene Transfer, and Founder Flush

**DOI:** 10.3201/eid0907.020760

**Published:** 2003-07

**Authors:** Pallavi Garg, Antonia Aydanian, David Smith, J. Glenn Morris, G. Balakrish Nair, O. Colin Stine

**Affiliations:** * National Institute of Cholera and Enteric Diseases, Calcutta, India; † International Centre for Diarrheal Diseases Research, Dacca, Bangladesh; ‡ University of Maryland School of Medicine, Baltimore, Maryland, USA

**Keywords:** cholera, evolution, base sequence, DNA, bacterial, evolution, molecular sequence data, research

## Abstract

*Vibrio cholerae* in O-group 139 was first isolated in 1992 and by 1993 had been found throughout the Indian subcontinent. This epidemic expansion probably resulted from a single source after a lateral gene transfer (LGT) event that changed the serotype of an epidemic *V. cholerae* O1 El Tor strain to O139. However, some studies found substantial genetic diversity, perhaps caused by multiple origins. To further explore the relatedness of O139 strains, we analyzed nine sequenced loci from 96 isolates from patients at the Infectious Diseases Hospital, Calcutta, from 1992 to 2000. We found 64 novel alleles distributed among 51 sequence types. LGT events produced three times the number of nucleotide changes compared to mutation. In contrast to the traditional concept of epidemic spread of a homogeneous clone, the establishment of variant alleles generated by LGT during the rapid expansion of a clonal bacterial population may be a paradigm in infections and epidemics.

An epidemic of cholera began in Madras, India, in 1992 and within a year had spread across the Indian subcontinent, with cases numbering in the millions (*1*,*2*). *Vibrio cholerae* isolates from this epidemic had a previously unidentified serotype, subsequently designated as O139 Bengal (*1*,*2*). This new serotype appears to have resulted when a lateral gene transfer (LGT) event occurred that replaced the 22 kb of the *wbf* region (encoding the O1 antigen) of a seventh pandemic *V. cholerae* O1 El Tor strain with a 37-kb region encoding the O139 polysaccharide (*3*–*5*). The epidemic spread rapidly through all age groups, as persons with previous exposure to *V. cholerae* O1 were not immune to O139 infection. Since 1992, O139 strains have established endemicity in this geographic region and account for a variable percentage of cholera cases every year (*6*).

Genetic variation observed in O139 isolates has been attributed to many causes. Variation in restriction fragment length polymorphism (RFLP) analysis of rDNA genes (*7*) and in *recA* sequence (*8*) has been interpreted as evidence for multiple origins. Genetic variability in RFLP of the CTX element (*6*) has been attributed to phage-mediated recombination. Variation in antimicrobial susceptibility (*9*) has been attributed to plasmid exchange in response to selective pressure from drug use. The variation in pulsed-field gel electrophoresis (PFGE) analysis of genomic restriction fragments (*6*,*10*) has been attributed to point mutations. Multilocus sequence typing (MLST), which has been used in the evaluation of a number of other bacterial species (*11*–*14*), provides an alternative method for measuring genetic relatedness and has provided data for identifying both point mutations and LGT events (*14*). MLST has improved discriminatory power over PFGE in some cases, e.g., *Enterococcus* (*15*) and *Salmonella* (*16*); however, in the case of *Escherichia coli* O157, it does not because of an absence of sequence variation in the clonally derived isolates (*17*). A small MLST study of O139 isolates of *V. cholerae* did not identify any LGT events (*18*).

To understand the evolutionary dynamics of *V. cholerae* O139, we sequenced segments from nine loci, including seven that may be classified as traditional housekeeping genes, one that carries the genes for cholera toxin, and another that is next to the insertion sequence within the O139 *wbf* region (*3*–*5*). Thus, the last two loci might be expected to show LGT, because they are associated with known mobile elements, but the other seven loci would not be expected to show LGT. However, we found putative LGT alleles at all nine loci in the 96 clonally related O139 isolates.

## Materials and Methods

We evaluated nine loci—*dnaE, lap, recA, pgm, gyrB, cat, chi, rstR,* and *gmd—*from 96 *V. cholerae* O139 isolated from patients seen at the Infectious Diseases Hospital, Calcutta, from 1992 to 2000 (see Appendix, online only). DNA was prepared from overnight cultures by using PrepMan Ultra (Applied Biosystems Inc., Foster City, CA) at the University of Maryland School of Medicine. Each locus was amplified by using polymerase chain reaction (PCR) with primers (Table 1) selected from a conserved region of the locus, as determined by aligning sequences from GenBank. Our primers selectively amplified the original O139 *rstR* gene found in all isolates and not the additional one found in some recently inserted CTX elements (*19*). The presence of amplified products was confirmed on agarose gels. Purification of the products was performed by using Millipore filters. The purified PCR products were sequenced in both directions by using the same primers used for amplification and Big Dye cycle sequencing kit (ABI) in accordance with manufacturer’s instructions. The fluorescently labeled products were separated and detected by using either an ABI 377 or 3700 Automatic Sequencer (ABI). The trace files were read by using Phred (*20*,*21*) and Phrap (*22*). Low-quality sequence at the ends was trimmed, and the contigs from each individual isolate were aligned by using Clustal X (*23*). Variable nucleotides were identified manually. Isolates with identical alleles were identified from a distance matrix obtained from PAUP (*24*). The alleles have been assigned GenBank accession numbers AY297845 to AY297921.

**Table 1:** Primers used for multilocus sequence typing

Locus	Primer 1	Primer 2
*dnaE*	CGRATMACCGCTTTCGCCG	GAKATGTGTGAGCTGTTTGC
*lap*	GAAGAGGTCGGTTTGCGAGG	GTTTGAATGGTGAGCGGTTTGCT
*rstR*	CGTGTTAGAGCACAC	GAGTGAATCGTCGTG
*gmd*	CCTTATGCKGTGGCRAA	CTWGGATCACCTAACA
*recA*	GAAACCATTTCGACCGGTTC	CCGTTATAGCTGTACCAAGCGCCC
*pgm*	CCKTCSCAYAACCCGCC	TCRACRAACCATTTGAADCC
*gyrB*	GAAGGBGGTATTCAAGC	GAGTCACCCTCCACWATGTA
*cat*	ATGGCTTATGAATCGATGGG	TCCCATTGCCATGCACC
*chi*	CAYGAYCCRTGGGCWGC	ACRTCTTCAATCTTGTC

**Figure 1:**
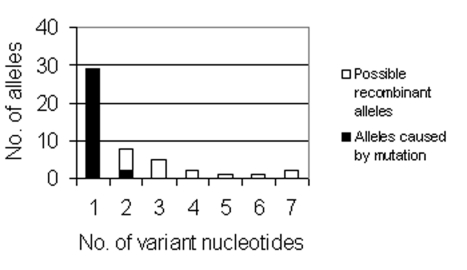
Bar graph of the number of novel alleles (y-axis) with a specific number of nucleotide differences from the ancestral allele. Two alleles with 24-bp and 113-bp differences are excluded from the graph.

The expected number of alleles that were a result of point mutations was calculated. All point mutations were assumed to occur independently; thus, the expected number of alleles with >2 nucleotides (nt) can be calculated, and the excess number of observed alleles was attributed to conspecific LGT of homologous genes. If one assumes that p is the probability of seeing a single mutation in an allele, the chance of seeing two mutations on the same allele is p^2^; the probability of seeing three or more mutations is p^3^. Probability can be calculated from the data by dividing the number of alleles with a single nucleotide difference, 34, by 785, the number of alleles in which observing a point mutation is possible (the 6-bp deletion; the recombinant *gmd,* *recA* alleles; and all duplicate novel alleles were excluded). Thus, p equals 0.043, p^2^ equals 0.0018, and p^3^ equals 8 x 10^-5^. When these probabilities are multiplied by the total number of alleles, 785, the expected number of alleles containing two independent point mutations is 1.45, and the expected number containing three or more is 0.06 (Figure 1).

## Results

Each of the loci examined had a variable number of observed alleles: 9 for *dnaE*, 20 for *lap*, 11 for *rstR*, 11 for *gmd*, 2 for *recA,* 8 for *pgm*, 4 for *gyrB*, 7 for *cat,* and 5 for *chi*. The most variable, *lap* with 20 alleles, was expected because it is a highly variable locus when analyzed with multilocus enzyme electrophoresis (*25*). The most common allele was present in 91% of isolates (n=87) for *dnaE*, 77% (n=86) for *lap*, 79% (n=90) for *rstR*, 82% (n=87) for *gmd*, 99% (n=96) for *recA,* 90% (n=94) for *pgm*, 97% (n=92) for *gyrB*, 93% (n=88) for *cat,* and 94% (n=89) for *chi*. Thus, the pattern for each locus consists of a common or ancestral allele and a series of rare alleles, as expected for the expansion of a clone.

Of the 64 less frequent alleles, some result from LGT and others from mutation. The three alleles with the largest changes are unlikely to be due to point mutations. First, a *gmd* allele that differed by 113 of the 360 bp sequenced, when compared with sequences in GenBank using BLAST (available from: URL: www.ncbi.nlm.nih.gov/BLAST/) showed greater similarity to *gmd* from *E. coli* (AF061251) than *gmd* from *V. cholerae,* consistent with LGT of a homologous gene into the *V. cholerae* genome. Second, an alternative *recA* allele that differs by 24 nt is likely to be the result of LGT of a homologous gene. Although substantial, the number of nucleotide differences is not large enough for the allele to be clustered with sequences from *V. mimicus*, the closest sibling species to *V. cholerae* (*8*), a finding that suggests that recombination occurred within *V. cholerae.* Third, a *lap* allele had a 6-bp deletion and a single nucleotide difference that may be the result of a double-strand break repair.

We calculated that at least 26 putative conspecific LGT events occurred in the 96 isolates studied. Figure 1 shows the number of nucleotide differences between each novel allele and the ancestral allele. If all point mutations are assumed to occur independently, the expected number of alleles with two or more variable nucleotides can be calculated and the excess number of observed alleles attributed to conspecific LGT of homologous genes. If all point mutations are assumed to occur independently, the expected number of alleles with two or more variable nucleotides can be calculated and the excess number of observed alleles attributed to conspecific recombination of homologous genes. The expected number of alleles containing two independent point mutations is 1.45, and the expected number containing three or more is 0.06. Since 11 alleles were observed with 2 nt differences, 9 more than expected, and 16 were observed with >3 differences, 16 more than expected, all of these alleles probably did not occur through mutation; more likely, these alleles are the result of LGT. Thus, we would estimate that 26 alleles (9 + 16 + *recA* allele above) are putatively due to conspecific LGT of homologous genes.

The putative conspecific LGT alleles, although fewer in number (26 alleles) than the assumed number of mutation-derived alleles (34 alleles), provide most of the nucleotide differences between alleles. The 120 nt changes introduced by conspecific LGT events are approximately three times the 38 (34 single mutations + 4 2x2 double mutations) introduced by mutation. This calculation is conservative: The 26 conspecific LGT events may represent an underestimate of the number because some of the alleles differing by <1 nt may have resulted from LGT.

The analysis of all nine loci from each isolate was based on the sequence type (ST). Each isolate was defined by a 9-digit number composed of the assigned allele number at each of the nine loci in the following order: *dnaE*, *lap*, *rstR*, *gmd,* *recA, pgm, gyrB, cat,* and *chi*. The most common allele was arbitrarily assigned as number 1. Thus, the ST of all the most common alleles is ST 1,1,1,1,1,1,1,1,1. Missing data were assigned the most common allele. This assumption is conservative, minimizes the observed amount of variation, and is consistent with the preponderance of common alleles found at each locus.

Fifty-one unique STs were found in the 96 isolates tested, reflecting relatively extensive genetic diversity. The overall average of 0.53 unique STs per isolate examined is similar to that seen in every year including 1992 (Table 2). Six STs occur more than once. As expected, the ancestral ST:1,1,1,1,1,1,1,1,1, found in 40 isolates, occurred in all years. Among the others, ST:1,1,2,1,1,1,1,1,1 was found three times, once each in 1995, 1996, and 1997. ST:1,2,1,1,1,1,1,1,1 and ST:1,1,7,1,1,1,1,1,1 were found twice in 1992 and 1994, respectively. ST 1,1,1,6,1,1,1,1,1 was found once in 1998 and again in 1999. ST:1,1,1,1,1,4,1,1,1 was found in 1995 and 1998. Since the number of STs is large (51 types), and number of samples in a collection period is small (8–13 samples; Table 2), STs seen in multiple years must not only persist but also represent a substantial portion of the epidemic O139 *V. cholerae* population.

**Table 2:** Number of isolates tested and distinct sequence types, by year

No.	1992	1993	1994	1995	1996	1997	1998	1999	2000	Total
Isolates examined	9	9	12	10	13	11	12	12	8	96
Novel sequence types	3	6	6	7	8	6	7	4	7	51
Novel sequence types per isolate examined	0.33	0.66	0.5	0.7	0.62	0.55	0.58	0.33	0.88	0.53

**Figure 2:**
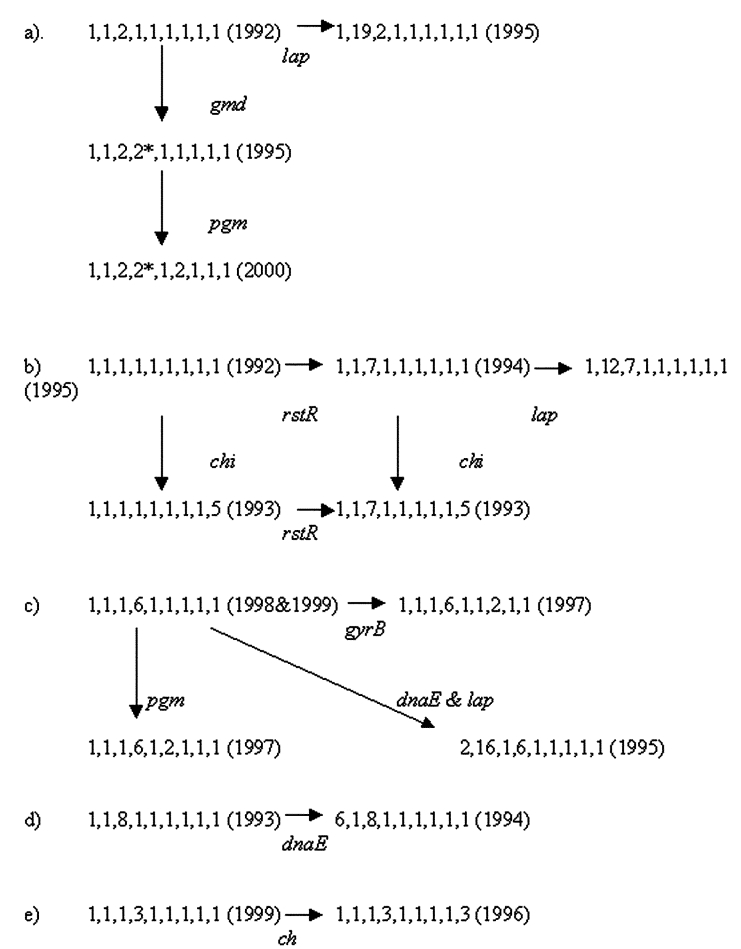
Five groups of related sequence types of *Vibrio cholerae* O139.

Five of the novel STs are related to other novel STs by allelic change at another second or third locus. One sequence type evolved into three related types found in subsequent years (Figure 2a). The starred *gmd* allele is one related to the *E. coli* sequence, and its presence in two distinct related STs in two different years demonstrates its establishment in the population. That the pattern seen in Figure 2b of ancestral alleles *rstR* 1 and *chi* 1 and two variant alleles, *rstR* 7 and *chi* 5, was found in all combinations is indicative of an LGT event. Figure 2c-e shows three additional groups of related sequences. In Figure 2a, b, and d, the ST with the larger number of novel alleles occurred in later years. In contrast, in Figure 2c and e, the ST with the larger number of novel alleles occurred in the earlier years. The lack of an overall temporal relationship may result from the small sample size (8–13 isolates) in any year.

One isolate, CRC5, is unusual because it has no sequenced alleles of the ancestral type. Nevertheless, the alleles from this isolate are closely related to those of the ancestral type. Each CRC5 allele differs from the ancestral allele by 7 nt for *dnaE*, 3 nt for *lap*, 4 nt for *rstR*, 24 nt for *recA*, 6 nt for *pgm*, 10 nt for *gyrB*, and 4nt for *chi*. A comprehensive survey of the genetic distances for these loci could determine the average distance between alleles for each of these loci. The data would provide insight into whether this isolate represents a second derivation of the O139 clinical type from an environmental strain or if it is a genetic outlier within the clonally related, but diversified, O139 epidemic type.

## Discussion

The emergence and pandemic spread of *V. cholerae* O139 Bengal represented a chance to examine evolution of a bacterial strain in the midst of a clonal expansion. Our results are consistent with clonal expansion and subsequent divergence as described by Spratt and Maiden (*26*). Putative recombinant alleles were found at all nine loci among the 96 clonally related O139 isolates. One *gmd* allele from *V. cholerae* was most similar to a *gmd* allele from *E.* *coli*. The number of base-pair differences among other alleles was higher than expected on the basis of a simple computation for the accumulation of independent mutations. This finding suggests that many of these events were due to LGT. When we applied our criteria to the novel alleles identified in a previous study (*18*), 11 of the 13 would be considered to have resulted from LGT, since the number of nucleotide differences to the ancestral allele varied from 4 to 19. Thus, for *V. cholerae*, like *Neisseria*, *Streptococcus*, and other bacterial species (*11*–*14*), conspecific recombination of homologous genes appears to be common and responsible for most of the alleles with multiple nucleotide differences and the majority of the nucleotide differences. The genetic variability at the nine loci alters our understanding of evolution in bacteria, showing that recombination in *V. cholerae* occurs frequently and most nucleotide changes occur by means of a recombination that can alter any gene.

The proportion of recombinants from conspecific recombination, 3.5% (28/785) is greater than that from transgeneric recombination (0.01% from the acquisition of *E. coli* *gmd* by one isolate). One potential implication of a greater rate of conspecific recombination may be that, over time, it will maintain the species identity of each individual bacterium, despite the constant bombardment of homologous genes from other genera. Although at first glance the frequency of the novel sequence types appears to conflict between our study and an earlier study (*18*), the observations may be reconciled on the basis of both the observed frequencies and the timing of the observations. Both studies identified a common ancestral allele in from 77% to 99% of isolates in our study and a series of rare alleles with 1–19 variant alleles for each locus. These studies reported 10% novel sequence types in 29 isolates that were collected from “the first epidemic period,” from 1992 to 1993 (*18*). Our data from 1992 showed 33% novel sequence types from a sample of nine. These data are not statistically different (chi-square test=2.4, p=0.12). However, the researchers’ estimate of frequency (*18*) is more likely to be correct because of the larger sample size. The dates of collection may also be important because our collection of isolates from 1993 began in March, when the number of O139 cases at the Infectious Diseases Hospital rose from <10 to >80 per month, corresponding to a rapid population expansion or flush. Thus, we can predict that we would see substantial variation in our sample.

The genetic diversity was greater in the *V. cholerae* O139 isolates than in other clinically associated clones. In *V. parahaemolyticus* O3:K6, a pandemic strain, 94% of strains were identical at four loci (N. Chowdhury et al., unpub. data). In *E. coli* O157, all 77 isolates were identical at seven loci in spite of variation between isolates on PFGE (*17*). Although *V. parahaemolyticus* and *E. coli* are widespread pathogens, they differ from *V. cholerae* O139 because their population size has expanded much more slowly.

Among O139 isolates, the substantial genetic diversity found in the first year of the epidemic may reflect a “founder flush” phenomenon. During times of population expansion, i.e., a flush, any novel genotype with similar or even slightly deleterious fitness compared to the founder genotype will produce sufficient offspring to become established in the population (*27*). A founder flush appears to have occurred in the establishment of *Helicobacter pylori* in a single person (*28*). Although other previous descriptions of this phenomenon have been limited to insects, specifically butterflies (*29*) and drosophilids (*30*), we believe that the founder flush phenomenon may become the paradigm for epidemic bacterial expansion in individual patients and populations. This founder flush phenomenon, in turn, has implications for our interpretation of “clonality” among epidemic isolates and for our understanding of factors that contribute to the emergence of new pathogenic strains.
